# Evaluation of Implementing TOM: A Group-Based Fall Prevention Programme among Community-Dwelling Older Adults in The Netherlands

**DOI:** 10.3390/ijerph18126360

**Published:** 2021-06-11

**Authors:** Sanne W. T. Frazer, Rozan van der Veen, Anneloes Baan, Mariëlle E. W. Hermans, Branko F. Olij

**Affiliations:** Consumer Safety Institute (VeiligheidNL), Overschiestraat 65, 1065 XD Amsterdam, The Netherlands; r.vanderveen@veiligheid.nl (R.v.d.V.); a.baan@veiligheid.nl (A.B.); m.hermans@veiligheid.nl (M.E.W.H.); b.olij@veiligheid.nl (B.F.O.)

**Keywords:** accidental falls, aged, prevention, health, independent living, physical functioning, quality of life, implementation science

## Abstract

There is strong evidence that effective fall prevention elements exist, but the implementation into society remains difficult. The aim of the current study is to describe and evaluate the implementation of the fall prevention programme “Thuis Onbezorgd Mobiel” (TOM). This novel approach combines effective components into a multidisciplinary group-based programme for adults aged 65 years or older with an increased risk of falling. To investigate the impact on several health-related outcomes such as subjective health, quality of life, physical functioning, and falls, we applied a quasi-experimental pre–post design including a follow-up period. A total of 164 older adults subscribed to the programme: 80 were eligible to start and 73 completed it. The impact analysis revealed a significant improvement in subjective health, physical functioning, and quality of life directly after participating in the programme. The impact on subjective health and quality of life persisted six months after the programme. Important facilitators for the implementation of the programme were social contact and clear communication. Lack of a concrete follow-up was seen as an important barrier. The results of the current research help guide further implementation of effective fall prevention interventions in practice.

## 1. Introduction

Approximately one-third of older adults aged 65 years or older fall once a year [1]. Ten percent of these falls are so serious that a visit to the emergency department is needed [1]. Furthermore, the mortality rate is high: in 2017, falls were the leading cause of unintentional injury deaths [2]. Prognosis shows that the number of fall-related injuries will increase in the upcoming years [3].

A fall can significantly impact the quality of life of an older adult [4], and may trigger a cascade of events [1,5]. Older adults tend to reduce their level of physical activity due to a fear of falling [6]. This can cause other health-related problems such as cardiovascular disease or diabetes, which in turn can decrease daily activity and physical functioning further, increasing the risk of a subsequent fall. Ultimately, it can lead to a decrease in self-management and independence, as there is a significant association between falls and loneliness, social isolation, and living alone [7].

Because the consequences of a fall can have a significant impact on the quality of life of an individual [8], prevention is essential. A review of Hopewell et al. (2018) concluded that multicomponent interventions, which usually include exercise, may reduce the fall rate and risk compared to usual care [9]. Sherrington et al. (2019) showed that an exercise intervention can reduce the rate of falls by 23 percent [1]. Furthermore, if the exercise intervention consists of balance and functional exercise combined with resistance training, the rate of falls reduces by 34 percent [1].

Even though there is strong evidence that effective elements exist regarding fall prevention, the implementation into practice remains difficult [10,11]. Healthcare professionals struggle to implement fall prevention interventions due to a lack of time, lack of finances, or lack of knowledge [3]. Therefore, it is important to study the impact of fall prevention in a real-world setting in which the intervention is implemented in a community setting tailored to the individual [10,11]. Furthermore, it is important to raise awareness about the risk of falling among older adults, as they are often not aware of their own risk and the measures they can take to prevent a fall [12].

In 2018, the fall prevention programme “Thuis Onbezorgd Mobiel” (TOM) was launched by healthcare researchers, nutrition counsellors, implementation experts, and an insurance company in consultation with healthcare professionals such as physiotherapists and dieticians. The objective of TOM is to support older adults living at home to increase their mobility and independence by reducing the number of falls. The programme focuses on raising awareness of the risk of falls, improving balance, and increasing knowledge on healthy nutrition and fall prevention. Furthermore, it focuses on the collaboration with local partners, as well as sustainable implementation in the immediate and long-term future.

TOM has a novel approach of combining specific components of fall prevention interventions in a single and structured multidisciplinary group-based programme which is adapted to a community setting. The current paper aims to describe the implementation and evaluation of the TOM programme in a neighbourhood in Rotterdam, the Netherlands. Descriptive studies, such as the current paper, are essential in order to understand a large-scale implementation [13]. The evaluation of TOM could be helpful to implement this and other fall prevention programmes on a larger scale.

## 2. Materials and Methods

TOM is a 14-week group-based fall prevention programme offered to community-dwelling older adults aged 65 years or over with an increased risk of falling. We first describe the components of the programme, then describe the study design, and finally describe the programme evaluation.

### 2.1. Phases and Components of the Programme

**Figure 1:**
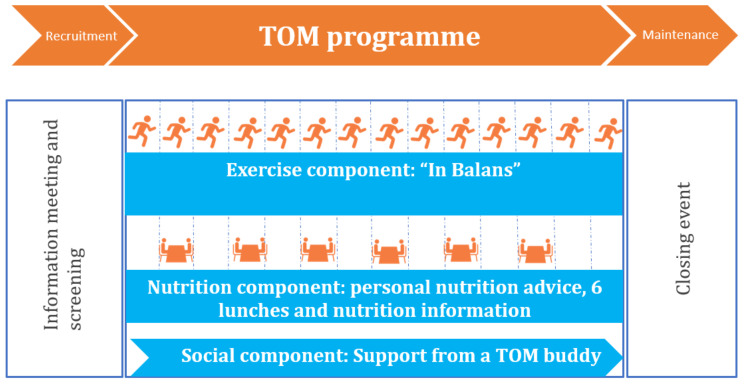
Phases of the “Thuis Onbezorgd Mobiel” (TOM) programme.

The TOM programme consists of three phases: (1) a recruitment phase including an information meeting and screening; (2) the main programme, consisting of an exercise, nutrition, and social component; and (3) a maintenance phase with a closing event (see Figure 1). In Rotterdam, the programme was supervised and led by two local coordinators. It was held in a local community centre and a local church.

#### 2.1.1. Information Meeting and Screening

The first phase consisted of an information meeting and screening. Older adults were recruited in different ways (see Section 2.3). Those who were interested attended the information meeting where professionals presented and explained the TOM programme. After the presentation, the older adults were screened by physiotherapists and dieticians on the basis of the selection criteria. The older adults who were excluded were directed to other programmes: the robust older adults were directed to other general exercise and health promotion programmes in the neighbourhood. The older adults that were too frail were directed to the appropriate professional to participate in an individual programme.

#### 2.1.2. Exercise Component

The exercise component in TOM consisted of the “In Balans” programme [14]. “In Balans” is based on Tai Chi and designed to improve general fitness, mobility, muscle strength, and balance. The length, duration, and content of the programme is in line with elements that are proven effective for fall prevention as stated in the systematic reviews of Sherrington et al. (2019) and Hopewell et al. (2018) [1,9]. “In Balans” is recognised as a proper substantiated programme by the CGL/RIVM lifestyle interventions committee for the Netherlands [15]. It is a 14-week group-based programme. Each group consisted of 10–12 participants guided by one physiotherapist. In the first three weeks, the older adults participated in one session per week in which they received information about falls and fall risk factors. Hereafter, they participated in training sessions twice a week for a period of 11 weeks to improve muscle strength and balance.

#### 2.1.3. Nutrition Component

The nutrition component consisted of two subcomponents. The first subcomponent was personal nutrition advice that was offered by a dietician and was based on a two-day food diary. The diary was filled in by the participants at the beginning and end of TOM. The second subcomponent consisted of nutrition information that was offered by a dietician during six group lunches centred around different themes, such as healthy eating, breakfast, and snacks. The lunches were offered before or after an “In Balans” session.

#### 2.1.4. Social Component

TOM was offered to participants in a group, facilitating possible new social contacts. Furthermore, personal support was offered by a trained volunteer, a so-called “TOM buddy”. The TOM buddy’s main tasks was to listen to, support, and motivate the participants throughout the programme. Each group was supported by one TOM buddy. In addition, they helped prepare the lunch and, if possible, supported the professional with their tasks.

#### 2.1.5. Closing Event

After 14 weeks, a closing event was organised in which participants and professionals reflected on the programme together. Participants attended a “market” of local exercise initiatives to see what would be appropriate for them after finishing TOM, in order to maintain their new skills (related to exercise, nutrition, and social contacts). Participants received tips from professionals on maintaining their exercise and healthy eating behaviour, as well as a booklet with information; tips; and an overview of the exercise, nutrition, and welfare programs in the neighbourhood.

### 2.2. Study Design

**Figure 2:**
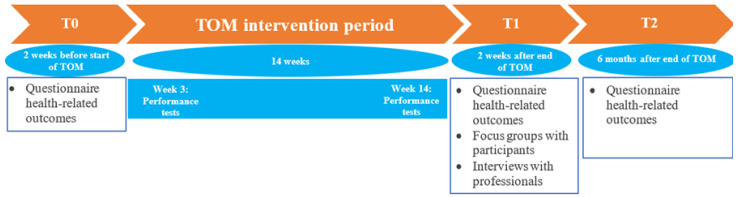
Study design of the current research. T0 = measurements before the start of the programme; T1 = measurements two weeks after the end of the programme; and T2 = measurements six months after the end of the programme.

The current research had a quasi-experimental pre–post design, including a follow-up period (Figure 2). To investigate the impact and implementation of TOM, we planned three measurements: T0 = before the start of the programme; T1 = two weeks after the end of the programme; and T2 = six months after the end of the programme. The recruitment of participants started in January 2019, and screening was conducted in February 2019. The programme started in April 2019 and lasted until July 2019. The last measurements for this study were executed in January 2020. All participants signed an informed consent form. The medical ethics committee of the University Medical Center Utrecht waived ethical approval of the study (18/693). In the current paper, results of the nutritional status of participants are not included, as this information is partly published [16] and will be published in a future paper.

### 2.3. Programme Evaluation

To evaluate the implementation of TOM, we have described key steps which are important for the implementation and maintenance of such a programme in a real-world setting. These steps are recruitment, impact on several health-related outcomes, and barriers and facilitators of implementation.

#### 2.3.1. Recruitment of Participants

This study was conducted in a neighbourhood in the city of Rotterdam, the Netherlands. On average, 15% of the population of Rotterdam is aged 65 years or older. In the neighbourhood of Rotterdam that was studied in this research, this percentage was higher, namely, 23% [17]. This was a reason to conduct the programme in this area. Older adults could join the programme if they were 65 or older or turned 65 in the year the programme started. To reach potential participants, we used several recruitment methods. Older adults were recruited to attend the information meeting via a press release and through advertisements in the local papers. Leaflets and a subscription box were displayed in local shops, the local church, and at the pharmacy in the neighbourhood. Additionally, word-of-mouth resulted in recruitment. At the local market, leaflets about the programme and a demonstration of some exercises were given by a Dutch TV personality. Furthermore, local professionals, such as the general practitioner, physiotherapist, social worker, and dietician, recruited participants during regular care.

During the information meeting, a screening was held to select eligible older adults. A physiotherapist used the Performance-Oriented Mobility Assessment (POMA) to test balance and physical performance of the older adult [18,19]. Along with the POMA, the 2-Minute Walk Test was executed [20,21]. If older adults scored below the cut-off score of 19 points on the POMA, they were advised to contact a physiotherapist to follow an individual programme. If older adults had a maximum score on the POMA, they were regarded as robust and were advised to join a regular exercise programme. During the screening, the Short Nutritional Assessment Questionnaire for 65+ (SNAQ-65+) was administered by a dietician to check for malnutrition [22].

The number of older adults who subscribed to and participated in the programme were collected. Characteristics of the participants, such as age, gender, education level, and living situation, were collected through the questionnaire that was administered at T0.

#### 2.3.2. Impact

We measured the impact of the programme on several health-related outcomes through questionnaires at T0, T1, and T2.

Subjective health was measured twofold: firstly, by asking about the participant’s current health status compared to the previous measurements, and secondly, by ranking their current health status on a Visual Analogue Scale (VAS) thermometer. Scores ranged from 0 to 100, in which 100 is the best health status possible.

The SF-36 physical functioning scale assessed the physical functioning of the participant [23], and the five-dimensional EuroQol instrument (EQ-5D) measured the generic quality of life [24].

Number of falls were assessed by self-reported falls over a period of six months before T0, the period between start and end of the programme (between T0 and T1, i.e., 4 months) and six months after the end of the programme (between T1 and T2), while the Short Falls Efficacy Scale-International (short FES-I) assessed the concern with falling [25].

Furthermore, several performance tests were used to measure physical functioning objectively in weeks 3 and 14 of the intervention period at the location where the exercise sessions were held. The Timed Up-and-Go test measured mobility and dynamic balance by measuring the time in seconds it took to get up from a chair, walk 3 m, turn, and sit back in the chair [26,27]. The reach test measured stability and static balance by measuring the distance in centimetres of how far an individual can reach forward with their arms horizontally without having to take a step [28]. The chair-stand test determined the strength of a participant by measuring the time in seconds it took to get up and sit down in a chair five times in a row [29]. Balance was measured using the four-balance test, consisting of four different positions: parallel stance, semi-parallel stance, tandem stance, and one-legged stance. Each position had to be maintained up to 10 s [30,31] (see Table A1 in Appendix A for a detailed overview of the measurements used).

Descriptive statistics were used to describe the impact on the health-related outcomes. Furthermore, for the within-group analyses, repeated measures ANOVA, and Student’s *t*-test were used to calculate differences T0 vs. T1, T0 vs. T2, and T1 vs T2. The analyses were performed using SPSS Statistical Data software (IBM, Armonk, NY, USA), version 25.

#### 2.3.3. Barriers and Facilitators of Implementation

To measure which factors contributed to and hindered a successful implementation of TOM, we performed a process evaluation after the end of the programme. This was examined through a questionnaire, two focus groups with participants, and semi-structured interviews with involved professionals. Furthermore, adherence to the programme and the efforts made for collaboration between professionals within the programme were described. Moreover, the costs of implementing the programme were calculated. Participants attended the programme free of charge. The costs of the programme were based on the number of enrolled participants. To determine the total costs of implementing TOM, we identified expenditures and categorised them into “preparation”, “delivery”, “closing activities”, and “organisation”.

## 3. Results

### 3.1. Rectruitment

**Figure 3:**
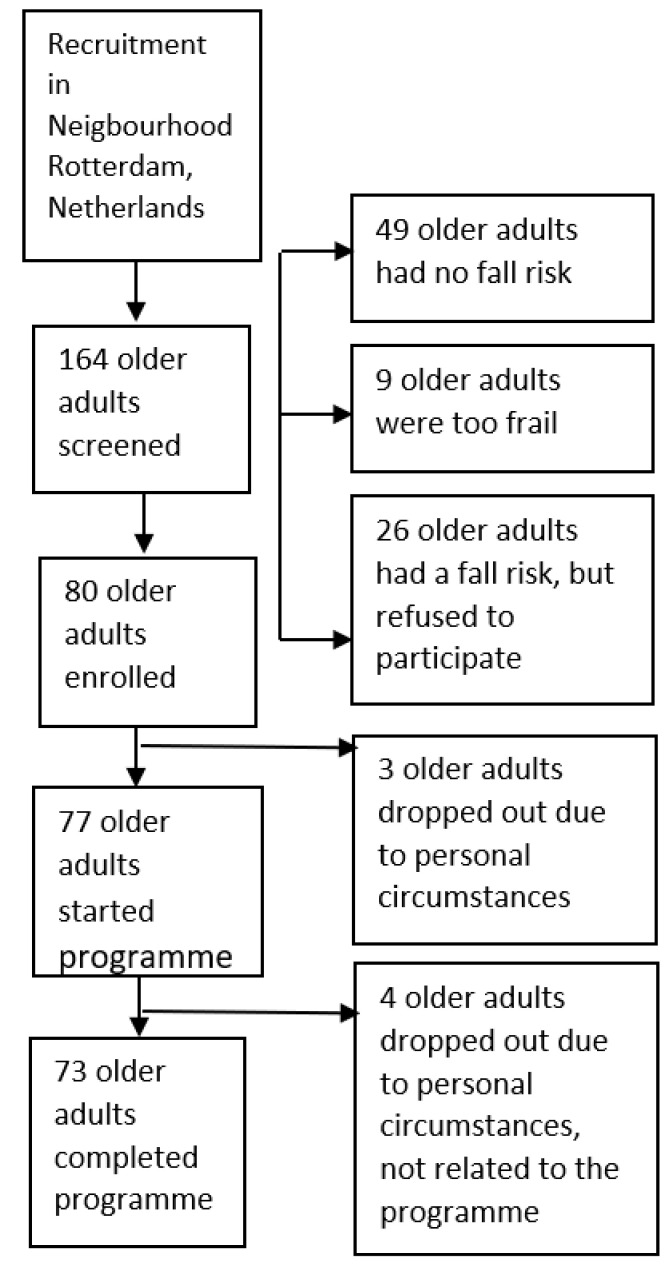
Flow-diagram of recruitment and reach of programme.

During the recruitment phase, 164 older adults subscribed to the programme. On the basis of the screening, 80 of them were eligible to start with the programme. A total of 77 older adults participated, and 73 completed the programme (Figure 3). Forty-six percent of the participants participated in all exercise sessions. Reasons for missing sessions were illness, holiday, (medical) appointments, funerals, or pain. A total of 67 percent of the participants attended all six lunches, 26 percent attended four to five lunches, and 7 percent attended two to three lunches.

Of the participants who enrolled, 58 individuals completed the questionnaire at all three measurements (i.e., T0, T1, and T2). This group comprised 12 men (20.7%) and 46 women (79.3%). The average age was 78.3 (±6.8) years (64–94 years) at the start of the programme. Just under 30 percent had completed junior general secondary education and a little over 25 percent had completed higher professional education. All participants lived independently, and 55 percent lived alone.

### 3.2. Impact

Table 1 shows the scores of the participants on the health-related measurements at T0, T1, and T2. The subjective health comparison increased significantly when comparing T0 vs. T1 (t(56) = 5.2, *p* < 0.05) and T0 vs. T2 (t(56) = 2.8, *p* < 0.05). However the subjective health comparison decreased significantly between T1 and T2 (t(57) = −2.7, *p* < 0.05), but remained superior to T0. The thermometer VAS score increased significantly when comparing T0 vs. T1 (t(57) = −3.88, *p* < 0.01) and T0 vs. T2 (t(57) = −2.64, *p* < 0.05). The thermometer VAS score did not significantly change between T1 and T2.

The average score on the SF-36 physical scale increased significantly when comparing T0 vs. T1 (t(39) = −2.435, *p* < 0.05). The SF-36 did not change significantly between T0 vs. T2 and T1 vs. T2.

The EQ-5D score significantly increased when comparing T0 vs. T1 (t(43) = −2.223, *p* < 0.05) and T0 vs. T2 (t(46) = −2.385, *p* < 0.05). The EQ-5D score did not significantly change T1 vs. T2.

Although there was not a statistically significant difference, the total number of reported falls (31 falls at T0) decreased to 24 falls at T1 and 16 falls at T2.

Of the 73 participants who finished the programme, 66 participants underwent the performance tests at both measurements in weeks 3 and 14. This group was on average 78.2 years (±6.9 years) old and comprised 15 men and 51 women. On average, the group improved significantly on tandem-stance (t(65) = −3.128, *p* < 0.05), one-legged stance (t(65) = −4.833, *p* < 0.05), the reach test (t(65) = −7.954, *p* < 0.05), the chair-stand test (t(64) = 7.464, *p* < 0.05), and the Timed Up-and-Go test (t(65) = 3.463, *p* < 0.05). In terms of percentages, when comparing the scores between weeks 3 and 14, 89 percent of the participants improved on strength, 78 percent improved on stability, 77 percent improved on dynamic balance, and 45 percent improved on static balance (Table 2).

### 3.3. Barriers and Facilitators of Implementation

Fifty-eight participants completed a process evaluation questionnaire. A total of 16 older adults (3 men, 13 women) participated during two focus groups, with an average age of 79 years. Ten professionals were interviewed by phone: four physiotherapists, two dieticians, two volunteers, and two local coordinators.

In terms of the questionnaire, the participants awarded the programme with an 8.6 out of 10, and 94 percent indicated that the goal of the programme was clear. The majority of the participants indicated that they had more social contacts, were more physically active, and felt fitter after participating in the programme.

Results of the focus groups and interviews indicated social contact and clear communication as facilitating factors. Participants experienced the group setting as a motivator to follow the programme and to exercise together. The programme was clearly explained. The transparency concerning the set-up of the programme and progress during the programme helped to increase the participants’ awareness of their own health and how to improve it. Furthermore, they appreciated the information materials as being understandable and practical. A barrier for the participants was the lack of a specific follow-up ready to continue with the same group immediately after the programme. The “market” of local exercise and food initiatives was expected to be a successful way to inform and help the participants to plan to maintain their health and well-being progress in other programmes. The offer of local exercise initiatives at the closing event was sufficient and varied. However, participants had a different view regarding the follow-up. They preferred a concrete and fixed follow-up programme, preferably with the same group of participants and professionals Another barrier was the duration of the programme; some participants considered the length of the programme and meetings to be too long and intense. Other facilitating factors and barriers are shown in Table 3.

Professionals indicated that during the execution of the programme, they learnt more about older adults with an increased fall risk and how to assist them. It gave them better opportunities to support the older population in the local area, which can be seen as a facilitating factor for the programme. A barrier for the professionals was the lack of collaboration between the dieticians and physiotherapists during the programme. It was advised to facilitate more moments of contact between dieticians and physiotherapists during the programme.

The total costs of the TOM programme were EUR 74,906, consisting of EUR 20,959 for preparation, EUR 38,794 for delivery, EUR 7557 for closing activities, and EUR 7597 for organisation.

## 4. Discussion

The current study describes the implementation of the TOM programme. The results showed that the participants experienced significantly better health after participating in the programme, which persisted during the six months after the end of the programme. The participants also improved significantly in terms of quality of life indicators at the end and six months after the end of the programme. The objective physical performance tests showed a significant improvement in strength, stability, and balance. The process evaluation revealed that participants appreciated the programme, that the goal of the programme was clear, and that half of the participants were planning to remain active. Furthermore, social contact and clear communication were facilitating factors for adoption and implementation of the programme. The lack of a concrete follow-up after the programme was a barrier for the implementation and maintenance of the programme.

The average quality of life scores of the TOM participants improved significantly over time (T0: 0.7578 (±0.17), T1: 0.7873 (±0.17), T2: 0.8123 (±0.16)). However, these scores are lower than the standard scores for the Netherlands, as reported by the EuroQol group (0.886 for 65–75 years and 0.839 for 75+ years) [24]. This could have been due to the composition of the group of participants; possibly more vulnerable older adults participated during the programme. Although the scores are lower than the standard scores, the scores did improve significantly after participating in TOM. The scores on the physical performance tests also improved. However, the clinical relevance was not investigated as cut-off scores for the performance tests strongly vary and a “gold standard” for fall risk is missing for the Dutch population.

The current recruitment methods yielded 164 interested older adults in the programme. This is 6.6 percent of all older adults of 65 years or older living in the neighbourhood where the programme was conducted [32]. The recruitment methods used in other studies, such as the advertisements in the local newspaper, leaflets in local shops, verbal recruitment at the local market, and recruitment by local professionals, also worked for the current study [33,34]. To increase the number of participants, a future recruitment method could be to email all inhabitants of 65 years or older in the neighbourhood. However, this is a high-cost recruitment method compared to other methods [34], and the privacy law in the Netherlands hinders easy access to contact information of all older adults in a particular area. Another recruitment method that could be applied is to make use of programme ambassadors. Former participants of the programme could be good ambassadors to promote the programme [34]. Even though we would like to reach all older adults in a neighbourhood with the TOM programme, not all older adults want to participate in a group. The study of Bartmentloo and colleagues showed that 46 percent of their participants preferred an individual programme versus 44 percent in a group [35]. Furthermore, to be a programme with impact, exercise and fitness levels have to fit the participant [1,35]. This already starts during the recruitment stage; if you do not recruit a participant that fits the programme, it will result in a non-effective and non-sustainable programme [36]. Therefore, a screening was incorporated in the recruitment phase of the TOM programme. Only those older adults who would benefit the most from participating in the programme were allowed to participate. Older adults who were excluded received information about fall prevention and other more appropriate programs or professional support. This made the excluded older adults more aware of their fall risk, resulting in the reach of prevention extending beyond the participants of the TOM programme.

Di Lorito concluded that group exercise also has other beneficial effects that go beyond the physical benefits, such as social integration and motivation [37]. As mentioned earlier, the current study showed these beneficial effects too, as the participants appreciated the group aspect, showed improvements on quality of life outcomes, and addressed the value of social interaction. Group cohesion helped participants to adhere to the programme, leading to a higher chance of beneficial health effects and a lower fall risk [35,38]. In a future study it would be interesting to study these aspects more objectively and investigate the social impacts on health and fall-related outcomes.

### Limitations

There are some limitations of the current study. Firstly, it is difficult to interpret the reduction in the number of falls participants experienced. By using a recall period of at least 4 months, we relied heavily on the memory of participants. Furthermore, at T0, T1, and T2, inconsistent recall times were used to collect the number of falls. This was due to a shorter intervention period (4 months) than recall period at T0 and T2 (6 months). To improve the reliability of this outcome, it is advised to use fall calendars instead of relying on self-reported falls over a longer period of time. Secondly, we do not know if the participants are a good representation of the population of older adults of the neighbourhood investigated. We did not take into account the migration background or socio-economic status of the participants. Older adults with lower socio-economic status and older adults with a migration background might have problems following the instructions of the programme [39]. Moreover, older adults with a migration background in general have a higher risk of falling [40,41]. Furthermore, we did not ask for other (chronic) health conditions. In a future study we need to take these factors into account to investigate their influence on the impact and suitability of the programme. Thirdly, it was not possible to investigate if there was a difference between included and excluded participants as we commenced data gathering after the screening process.

Furthermore, we used components already proven effective following the reviews of Sherrington et al. and Hopewell et al. [1,9]. Therefore, it was not the aim of the current study to prove effectiveness. However, we do emphasise here that the results on health-related outcomes must be taken with caution. We used self-reported methods to measure the impact. Furthermore, we did not perform a sample size calculation, and thus results could be underpowered. Another improvement could be to use an Randomised Controlled Trial (RCT) set-up to have a more solid way of measuring the variables with a matched control group. This will improve the outcomes of the study and will make it possible to evaluate the effectiveness of the programme properly.

Implementation research, such as the current study, is necessary in order to close the gap between theory and practice. Knowledge on effective fall prevention interventions exists in abundance, but it remains difficult to implement it properly. Factors such as investment in good collaboration between professionals and ownership of the programme are also two important facilitators for maintenance of a fall prevention programme. Furthermore, every local situation is different [42]; from the composition of older adults living in that particular area, to the availability and knowledge of local professionals. This demands adaptation in the implementation process. If barriers and facilitators are described well, more strategies can be developed to achieve a successful implementation in different situations [43]. In a future study, it would be useful to take the current limitations into account to gather more evidence about the successfulness of the programme itself and its implementation. To contribute to successful implementation strategies, the TOM consortium has written a manual to help professionals implement the TOM programme in their area [44].

## 5. Conclusions

This study describes the implementation of the TOM programme and showed promising results on subjective health belief, quality of life, stability, strength, and balance of older adults with an increased risk of falling. It also resulted in facilitating factors such as social contacts and clear communication, but also gave insight into barriers such as a lack of a clear follow-up. Lessons learned during this process are important for further dissemination of the programme in other areas and for other fall prevention interventions.

## Figures and Tables

**Table 1:** Scores on health-related measurements on T0, T1 and T2.

Self-Reported Health-Related Variables	T0	T1	T2
Self-reported health—av (SD)	3.16 (±0.75)	2.51 (±0.78) *	2.81 (±0.83) ^±, α^
Much better than previous measurement—% (#)	3.5% (2)	12.1% (7)	10.3% (6)
Somewhat better previous measurement—% (#)	8.8% (5)	32.8% (19)	17.2% (10)
Roughly the same as previous measurement—% (#)	57.9% (33)	51.7% (30)	58.6% (34)
Somewhat worse than previous measurement —% (#)	28.1% (16)	1.7% (1)	12.1% (7)
Much worse than previous measurement—% (#)	1.8% (1)	1.7% (1)	1.7% (1)
VAS score self-reported health—av (SD)	70.2 (±13.2) (range 40–95)	75.2 (±11.0) * (range 50–98)	73.8 (±11.0) ^±^ (range 45–100)
EQ5-DL total score—av (SD)	0.7578 (±0.17)	0.7873 (±0.17) *	0.8123 (±0.16) ^±^
SF-36 score—av (SD)	68.0 (±21.12)	72.5 (±21.4) *	65.8 (±24.3)
Short FES-I score—av SD	10.7 (±3.6)	10.3 (±3.3)	10.1 (±3.3)
Suffered a fall?			
Yes—% (#)	31.0% (18)	29.3% (17)	22.4% (13)
No—% (#)	69.0% (40)	70.7% (41)	77.6% (45)
Number of falls			
Fell once—#	7	10	10
Fell twice—#	8	8	3
Fell three times—#	12	6	
Fell four times—#	4		
Total number of falls—#	31	24	16
Per faller—falls/# fallers	1.94	1.5	1.2

T0 = measurements before the start of the programme; T1 = measurements two weeks after the end of the programme; T2 = measurements six months after the end of the programme; av = average; SD = standard deviation; % = percentage; # = number; VAS = Visual Analogue Scale; EQ5-DL = the five-dimensional EuroQol instrument; SF-36 = SF-36 physical functioning scale; short FES-I = Short Falls Efficacy Scale-International; * significant difference T0 vs. T1; ^±^ significant difference T0 vs. T2; ^α^ significant difference T1 vs. T2.

**Table 2:** Average scores for the performance tests.

Performance Test	Week 3	Week 14	*p*-Value	% Progress between Week 3 and 4 of Total Participants
Timed Up-and-Go (mobility/dynamic balance) (s) (*n* = 66)	9.9 s (±2.5)	8.8 (±3.0)	*p* < 0.01	77.3%
Reach test (stability/static balance) (cm) (*n* = 66)	21.3 cm (±5.9)	26.1 (±6.4)	*p* < 0.01	78.8%
Chair-stand test (strength) (s)(*n* = 65)	16.4 (±5.1)	12.4 (±3.8)	*p* < 0.01	89.2%
Balance; parallel stance (s) (*n* = 66)	10 (±0)	10 (±0)	--	--
Balance; semi-parallel stance (s) (*n* = 66)	9.7 (±1.6)	9.98 (±0.18)	*p* = 0.203	3.0% (95.5% same)
Balance; tandem stance (s) (*n* = 66)	8.7 (±3.0)	9.7 (±1.4)	*p* < 0.01	18.2% (80.3% same)
Balance; one-legged stance (s) (*n* = 66)	6.0 (±3.9)	7.7 (±3.3)	*p* < 0.01	45.5% (43.9% same)

**Table 3:** Facilitators and barriers of implementation.

Facilitators	Participants	Physiotherapists	Dieticians	Volunteers	Local Coordinator
Group dynamic/social contact	x	x	x	x	x
Clear communication	x	x	X	x	x
Visible effect	x	x	n.a.	n.a.	x
Set duration	x	n.a.	n.a.	x	n.a.
Acquiring knowledge/informative	x	n.a.	n.a.	x	x
Physical support	x	n.a.	n.a.	n.a.	n.a.
Not feeling under any obligation to take part	x	n.a.	n.a.	n.a.	n.a.
Organisational support from volunteer	n.a.	x	x	x	n.a.
Contact with clients in own neighbourhood	n.a.	x	n.a.	x	n.a.
**Barriers**					
Lack of follow-up after programme	x	x	x	n.a.	x
Groups at nutrition information session too big/hard to understand	x	n.a.	x	n.a.	n.a.
Costs/financing	x	x	x	n.a.	X
Difference in physical level	x	x	n.a.	n.a.	n.a.
Absence of participants	x	n.a.	n.a.	n.a.	n.a.
Lack of collaboration between physiotherapist/dietician	n.a.	x	x	n.a.	n.a.
Not enough visible effect	n.a.	n.a.	x	n.a.	n.a.
Lack of materials/proper timetable	n.a.	x	x	x	x
Big time investment	n.a.	n.a.	n.a.	x	x

x = applicable; n.a. = not applicable.

## Data Availability

The data presented in this study are available on request from the corresponding author. The data are not publicly available because they represent information that could compromise the privacy of the study participants.

## Appendix A

**Table A1:** Overview of measurements.

		Source	Baseline/Start (T0)	Post-Intervention/End (T1)	6 Months Follow-Up (T2)
Socio-demographic information	Age, gender, education, marital status, living situation	Self-report	x	x	x
Falls history	Number of falls past period	Self-report	x	x	x
Quantitative/impact measures					
Clinical performance-based measures		Physiotherapist			
Static balance	4-balance test [29,30]		x	x	
Dynamic balance/mobility	Timed Up-and-Go [25,26]		x	x	
Stability/static balance	Functional reach test (Duncan)		x	x	
Strength	Chair-stand test [28]		x	x	
Self-report					
Subjective health		Self-report	x	x	x
Fear of falling	FES-I [24]	Self-report	x	x	x
Quality of life	EQ-5D [23]	Self-report	x	x	x
Physical functioning	SF-36 pfs [22]	Self-report	x	x	x
Costs	Costs of preperation, delivery, closing activities, and organisation of the programme	Physiotherapists, dieticians, TOM buddy, local coordinators	x	x	
Programme acceptability					
Adherence		Self-report		x	
Satisfaction		Self-report		x	
Qualitative measures					
Participants	Focus group; barriers and facilitators			x	
Professionals	Semi-structured interviews; barriers and facilitators			x	
